# Pomalidomide for the Treatment of Multiple Myeloma

**DOI:** 10.6004/jadpro.2014.5.1.7

**Published:** 2014-01-01

**Authors:** Stephen M. Clark, Alison Steinbach, Amber B. Clemmons

**Affiliations:** From Georgia Regents Medical Center and University of Georgia College of Pharmacy, Augusta, Georgia

Multiple myeloma (MM) is a malignancy marked by proliferation and clonal expansion of plasma cells and excessive production of monoclonal immunoglobulin, which can lead to end-organ damage presenting as hypercalcemia, renal insufficiency, anemia, and/or bone lesions (Kyle, 2012). An estimated 22,350 new cases of MM were projected in the United States for 2013, including approximately 10,710 deaths (Siegel, Naishadham, & Jemal, 2013).

The use of autologous stem cell transplantation (ASCT) and novel agents such as the immunomodulatory drugs (IMiDs) thalidomide (Thalomid) and lenalidomide (Revlimid) as well as the proteasome inhibitors bortezomib (Velcade) and carfilzomib (Kyprolis) improved the estimated 5-year overall survival (OS) from 28.8% in the period between 1990 and 1992 to 34.7% in the period between 2002 and 2004; however, nearly all patients with MM will experience disease relapse (Brenner, Gondos, & Pulte, 2008; Richardson et al., 2013). Furthermore, patients who are refractory to or unable to receive thalidomide or lenalidomide have a median OS of only 9 months (Dimopoulos et al., 2012). See Table 1 for a comparison of immunomodulatory drugs pomalidomide, lenalidomide, and thalidomide.

**Table 1:**
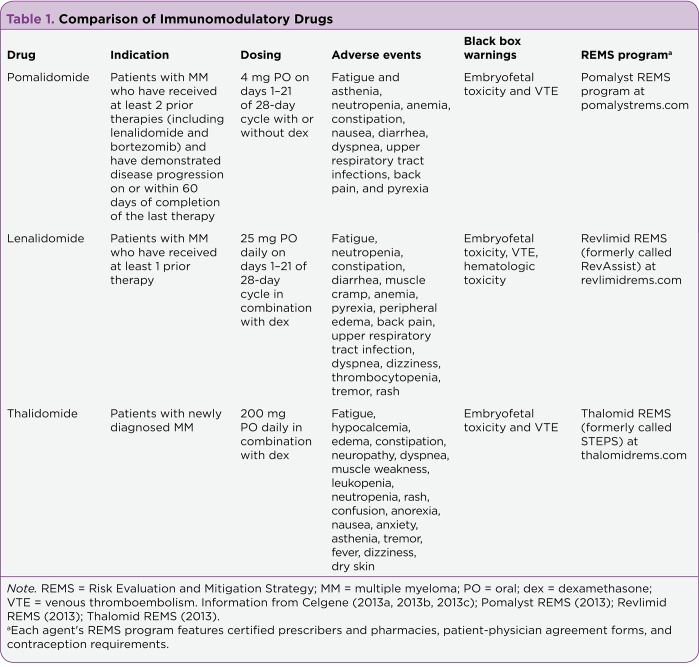
Table 1. Comparison of Immunomodulatory Drugs

The choice of therapy at the time of relapse is based on the duration of response to prior treatment and patient tolerability. In general, patients who experience relapse more than 6 months after the completion of treatment may be retreated with the previously effective agent or agents (Kyle, 2012). If a patient with MM relapses earlier than 6 months, a treatment regimen with alternative agents should be implemented (Palumbo et al., 2009; Palumbo & Anderson, 2009). In the setting of refractory disease, a change in treatment regimen is also warranted (Leleu et al., 2013).

Pomalidomide (Pomalyst) is a thalidomide analog immunomodulatory agent (Celgene, 2013a). On February 8, 2013, the US Food and Drug Administration (FDA) approved pomalidomide for the treatment of MM in patients who have received two or more prior therapies, including lenalidomide and bortezomib, and have demonstrated disease progression on or within 60 days of completion of their last therapy (FDA, 2013).

## Mechanism of Action

Pomalidomide is an IMiD that possesses antimyeloma properties including anti-inflammatory, antiangiogenic, antiproliferative, and immunomodulatory effects. The in vitro effects of IMiDs are diverse, and the exact mechanism of each corresponding effect to its anti-MM activity remains unclear. However, based on in vitro data, the most crucial mechanism of action of IMiDs is the disruption of MM–bone marrow stromal cell (BMSC) interactions. This results in the downregulation of crucial cytokines and growth factors necessary for myeloma growth.

Pomalidomide was derived from thalidomide by adding an amino group to the fourth carbon of the phthaloyl ring of thalidomide, resulting in increased potency of both anti-inflammatory and antiangiogenic properties with reduced toxicities (Quach et al., 2010). Compared with thalidomide, in vitro inhibition of tumor necrosis factor–alpha (TNFá) was greater with pomalidomide (Muller et al., 1999). TNFá is one of the proinflammatory cytokines that facilitate cancer development via modulation of cell adhesion interactions of myeloma cells with the tumor microenvironment. IMiDs downregulate cell adhesion molecules and reduce the secretion of interleukin (IL)-6 and vascular endothelial growth factor (VEGF), important growth factors for MM cell survival and proliferation. IMiDs also exhibit antiangiogenesis effects via the modulation of chemotactic factors (TNFá, VEGF, and â–fibroblast growth factor [âFGF]) involved in endothelial cell migration. IMiDs demonstrate antiproliferative effects by inducing apoptosis and blocking the upregulation of adhesion molecules on MM cells and BMSC.

The costimulatory effects on T cells and natural killer (NK) cells are another important property of IMiDs toward enhancing anti-MM immune activity. Pomalidomide appears to be more potent than both thalidomide and lenalidomide with regard to T-cell costimulation (Corral et al., 1999). Although treatment with IMiDs has translated into a significant extension of OS in MM, the mechanism of action of these agents as they relate to immune modulation is not fully known. In the future, designing optimal therapeutic combinations with these agents is contingent upon further understanding of their diverse mechanisms (Quach et al., 2010).

## Clinical Efficacy

The FDA approval of pomalidomide was based on a phase II, multicenter, randomized, open-label study. Patients with relapsed MM who were refractory to their last therapy and who had previously received lenalidomide and bortezomib were randomized to receive pomalidomide alone (n = 108) or pomalidomide with low-dose dexamethasone (n = 113). The pomalidomide regimen was 4 mg by mouth once daily for 21 days of a 28-day cycle. In the combination arm, in addition to pomalidomide, 40 mg of dexamethasone was given on days 1, 8, 15, and 22 of each cycle (although patients older than age 75 received 20 mg of dexamethasone). Most patients were heavily pretreated, with a median of five prior therapies in each arm. Furthermore, roughly 80% of patients had received prior autologous stem cell transplantation. The overall response rates (complete response plus partial response) were 7.4% (95% confidence interval [CI] = 3.3%–14.1%) and 33% (95% CI = 21.0%–38.5%) in the pomalidomide-alone arm and the combination arm, respectively (Celgene, 2013a).

In another randomized, phase II trial, 84 patients with relapsed MM were given dexamethasone 40 mg once weekly with pomalidomide 4 mg daily for 21 days (arm 21/28) or every day (arm 28/28) of a 28-day cycle (Leleu et al., 2013). The rate of partial response or better was 35% in arm 21/28 day and 34% in arm 28/28. Median progression-free survival (PFS) and OS were similar in both groups, with a total PFS of 4.6 months and a total OS of 14.9 months.

A phase III, multicenter, randomized, open-label study in patients with relapsed MM who were refractory to lenalidomide and bortezomib compared pomalidomide plus low-dose dexamethasone (POM + LoDEX) vs. high-dose dexamethasone (HiDEX) alone (Dimopoulos et al., 2012). A total of 455 patients were randomized 2:1 to POM + LoDEX or HiDEX. The POM + LoDEX arm received pomalidomide 4 mg daily for 21 days of a 28-day cycle with dexamethasone 40 mg once weekly (20 mg for patients over 75 years of age). The HiDEX arm received dexamethasone 40 mg (20 mg for patients over 75 years of age) on days 1–4, 9–12, and 17–20 of a 28-day cycle.

Median PFS was 15.7 weeks in the POM + LoDEX group vs. 8.0 weeks in the HiDEX group (*p* < .001). A planned interim OS analysis was significantly in favor of POM + LoDEX (median not reached vs. 34 weeks, *p* < .001), which crossed the prespecified O’Brien-Fleming superiority boundary.

## Dosing and Administration

The recommended starting dose of pomalidomide is 4 mg orally once daily on days 1–21 of a repeated 28-day cycle with or without dexamethasone (Celgene, 2013a). Dexamethasone is given at a dose of 40 mg orally once weekly; however, the dose of dexamethasone is reduced to 20 mg weekly for patients older than age 75. Cycles should be repeated until disease progression. To initiate a new cycle of pomalidomide, the patient’s neutrophil count must be ≥ 500/µL, and the platelet count must be ≥ 50,000/µL.

Pomalidomide and its metabolites are primarily excreted by the kidneys and metabolized by the liver; however, the impact of renal or liver impairment on the safety, efficacy, and pharmacokinetics has not yet been evaluated. Pomalidomide is not recommended in patients with a serum creatinine level > 3 mg/dL, a serum bilirubin level > 2 mg/dL, or aspartate transaminase and alanine transaminase levels greater than three times the upper limit of normal because these patients were excluded in clinical studies.

Specific dosing modifications for hematologic toxicities are listed in Table 2. For grade 3 or 4 nonhematologic toxicities, treatment should be held until the toxicity has resolved to a ≤ grade 2 reaction. Following grade 3 or 4 toxicity, the dose should be restarted at 1 mg less than the previous dose. If toxicities occur after the dose has been reduced to 1 mg, pomalidomide administration should be discontinued.

**Table 2:**
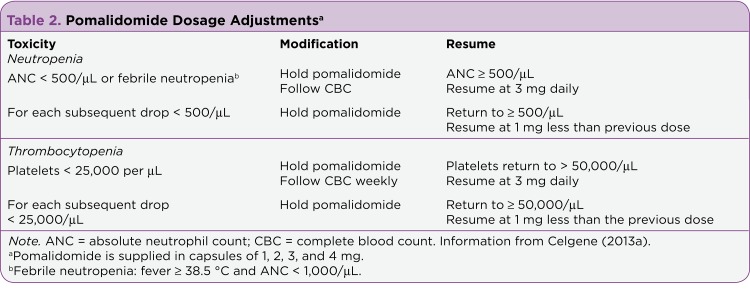
Table 2. Pomalidomide Dosage Adjustments

Pomalidomide should be administered on an empty stomach (at least 2 hours before or 2 hours after a meal). Missed doses may be administered if within 12 hours of the usual dosing time of pomalidomide. If more than 12 hours have elapsed following the last dose, the dose for that day should be skipped, and the usual dosing should be resumed the following day.

As pomalidomide is a pregnancy category X agent, it is contraindicated during pregnancy. Due to the embryofetal risk, pomalidomide is available only through a Risk Evaluation and Mitigation Strategy (REMS)–restricted distribution program. The goals of the REMS program are to prevent embryofetal exposure to pomalidomide and to inform prescribers, patients, and pharmacists about the serious risks and safe-use conditions for pomalidomide. Prescribers and dispensing pharmacies must be certified to participate in the Pomalyst REMS program. Patients must sign a patient-physician agreement form and comply with the REMS requirements. The REMS program includes contraceptive requirements for male and female patients and regular pregnancy testing for women of childbearing age who receive the drug (Pomalyst REMS, 2013).

## Adverse Events

In the phase II study, frequent adverse effects reported in the pomalidomide group (n = 107) included fatigue (55%), neutropenia (52%), anemia (38%), constipation (36%), nausea (36%), diarrhea (34%), back pain (32%), and upper respiratory tract infection (32%). Overall, grade 3/4 adverse reactions were comparable among the two groups (90% in the pomalidomide group vs. 88% in the POM + LoDEX group). As in other pomalidomide trials, grade 3/4 toxicity consisted primarily of myelosuppression in the form of neutropenia (47% vs. 38%), anemia (22% vs. 23%), thrombocytopenia (22% vs. 19%), and leukopenia (6% vs. 10%). According to the prescribing information for each drug, pomalidomide appears to be associated with less peripheral neuropathy than thalidomide (grade 3/4, 0% vs. 3% to 4%, respectively; Celgene, 2013a; 2013c).

Due to the risk of venous thromboembolism with pomalidomide, patients in clinical trials were required to receive prophylaxis or antithrombotic treatment. In the phase II study, 81% used aspirin, 21% used heparin, 16% used warfarin, and 3% used clopidogrel. The rate of deep vein thrombosis or pulmonary embolism was 3%. Pomalidomide carries a boxed warning for venous thromboembolism, and it is recommended to consider anticoagulation prophylaxis after an assessment of each patient’s underlying risk factors (Celgene, 2013a).

## The Role of Pomalidomide in Therapy

Based on current FDA labeling, pomalidomide is a third-line agent for patients with relapsed or refractory MM. Although several other salvage regimens exist, some patients may not be able to tolerate multiagent chemotherapy regimens (such as VTD-PACE [bortezomib, thalidomide, dexamethasone, cisplatin, doxorubicin, cyclophosphamide, etoposide] or DCEP [dexamethasone, cyclophosphamide, etoposide, cisplatin]) and/or may already be resistant or intolerant to other salvage regimens that incorporate lenalidomide and/or bortezomib. Further, conveniently administered oral regimens that allow for outpatient management are preferred. Pomalidomide offers an orally administered alternative for these patient populations who have failed to respond to prior therapies.

In addition to pomalidomide, another new agent has been approved for the treatment of relapsed and refractory MM. Carfilzomib is a proteasome inhibitor that is approved by the FDA for patients with disease progression within 60 days of therapy who have received at least two prior therapies including bortezomib and an IMiD.

The choice between pomalidomide and carfilzomib, among the other salvage options, should be based on each individual patient’s prior treatment regimens, tolerability, renal/hepatic function, and medical history. At this time, no specific guidelines delineate the order in which these salvage regimens should be utilized.

## Conclusion

Using current treatment modalities, almost all patients with MM will experience disease relapse (Brenner et al., 2008; Richardson et al., 2013). Patients with relapsed or refractory disease have a poor prognosis; however, the OS benefit demonstrated with the IMiD pomalidomide plus Lo-DEX vs. Hi-DEX is encouraging given this highly pretreated patient population (Dimopoulos et al., 2012). The approval of pomalidomide is a welcomed third-line treatment option for patients with relapsed and refractory MM after treatment regimens including lenalidomide and bortezomib (FDA, 2013).
